# Development and multicenter validation of a machine learning model for predicting in-hospital postoperative cardiac arrest in elderly surgical patients: the critical role of intraoperative hemodynamic disturbances and blood transfusion

**DOI:** 10.3389/fmed.2026.1922481

**Published:** 2026-10-13

**Authors:** Xi Chen, Yuwei Chang, Tao Sun, Yuan Liu, Lei Liu, Chao Jiang, Feng Zhan, Wenyi Du

**Affiliations:** 1 Department of Hepatobiliary Surgery, The Affiliated Yixing Hospital of Jiangsu University, Yixing, China; 2 Yixing Branch of the Wuxi Medical Centre Affiliated to Nanjing Medical University, Yixing, China; 3 Department of General Surgery, The Affiliated Tengzhou Central People’s Hospital of Xuzhou Medical University, Zao Zhuang, China; 4 Clinical Medical Center, Tengzhou Central People's Hospital, Tengzhou, Shandong, China; 5 Gaomi People's Hospital, Gaomi, Shandong, China; 6 Tengzhou Hospital of Traditional Chinese Medicine, Shandong, China

**Keywords:** cardiac arrest, external validation, inflammatory response, machine learning, predictive model

## Abstract

**Background:**

In-hospital cardiac arrest (IHCA) is a rare but life-threatening complication in elderly surgical patients. This study aimed to develop and validate a machine learning model for predicting IHCA and to identify key perioperative risk factors.

**Methods:**

A multicenter retrospective cohort study was conducted among patients aged ≥65 years who underwent non-cardiac surgery at six tertiary hospitals between January 2020 and January 2025. Clinical, laboratory, and perioperative variables were collected. Five machine learning algorithms, including Extreme Gradient Boosting (XGBoost), Random Forest (RF), Support Vector Machine (SVM), Multilayer Perceptron (MLP), and K-Nearest Neighbor (KNN), were developed and compared. Model performance was evaluated using receiver operating characteristic (ROC) analysis, calibration, decision curve analysis, cross-validation, and external validation. SHAP analysis was applied to interpret the optimal model.

**Results:**

Among the five models, XGBoost demonstrated the best predictive performance, with AUCs of 0.967 and 0.960 in the training and validation cohorts, respectively. It also achieved the lowest Brier score and the highest clinical net benefit. External validation confirmed excellent discrimination (AUC=0.971) and calibration. SHAP analysis identified advanced age, intraoperative tachycardia, intraoperative hypotension, intraoperative blood transfusion, intraoperative hypoxemia, elevated postoperative neutrophil-to-lymphocyte ratio, B-type natriuretic peptide, lactate, and D-dimer levels as the most important predictors of IHCA.

**Conclusion:**

An interpretable XGBoost-based model demonstrated excellent performance for predicting postoperative cardiac arrest in elderly patients. The identified risk factors reflected perioperative vulnerability, intraoperative hemodynamic instability, and postoperative physiological deterioration.

## Introduction

With the accelerating global ageing of the population and continuous advances in surgical and anaesthetic techniques, the number of elderly patients undergoing operative procedures has steadily increased. Owing to progressive organ dysfunction, diminished physiological reserve, and the high prevalence of multiple comorbidities, advanced age has been widely recognised as a major risk factor for adverse perioperative outcomes. Among the spectrum of perioperative complications, in-hospital cardiac arrest (IHCA) represents one of the most catastrophic events, carrying an exceedingly high mortality rate and substantially increasing hospital length of stay, healthcare resource utilisation, and socioeconomic burden (1–3). Accordingly, early identification of high-risk individuals and the implementation of precise preventive interventions are of critical clinical importance.

Ageing is accompanied by progressive cardiovascular remodeling and a reduction in physiological reserve, which may impair the ability to compensate for acute perioperative stress. Older adults have a higher prevalence of cardiovascular conditions and age-related abnormalities involving myocardial function, vascular compliance, and cardiac conduction, all of which may increase susceptibility to haemodynamic instability and myocardial injury during surgery (1, 4, 5). These pre-existing vulnerabilities may be further amplified by the substantial physiological stress imposed by non-cardiac surgery, including fluctuations in blood pressure and heart rate, impaired oxygenation, blood loss, transfusion, and tissue hypoperfusion. In patients with limited cardiovascular reserve, the accumulation of these insults may precipitate myocardial dysfunction, systemic physiological deterioration, and ultimately IHCA. Therefore, identifying elderly surgical patients who develop clinically relevant cardiovascular and systemic physiological disturbances during the perioperative period may provide an opportunity for earlier recognition of those at particularly high risk of IHCA and for targeted postoperative surveillance and management.

At present, conventional clinical risk stratification tools, including the Revised Cardiac Risk Index (RCRI) and the American Society of Anesthesiologists (ASA) physical status classification, are widely used for perioperative risk assessment (6–10). However, these tools were developed for broader perioperative or cardiovascular risk assessment rather than specifically for predicting IHCA. Their target outcomes and intended applications therefore do not directly correspond to IHCA, a relatively rare but catastrophic event that may arise from the interaction between baseline patient vulnerability and acute perioperative physiological disturbances. In addition, these conventional tools provide relatively generalized assessments of preoperative risk and may not adequately capture the specific risk profile of elderly patients undergoing non-cardiac surgery or the dynamic physiological changes that precede postoperative IHCA. Importantly, clinical scoring systems specifically designed and routinely used to predict postoperative IHCA remain limited in current practice. Therefore, an IHCA-specific prediction model that integrates patient vulnerability with perioperative physiological disturbances may provide more targeted risk stratification and facilitate the early identification of high-risk patients who may benefit from intensified postoperative surveillance and individualized management.

In recent years, machine learning approaches have demonstrated substantial promise in perioperative risk prediction (11–16). Compared with traditional statistical methods, machine learning can accommodate high-dimensional, nonlinear, and heterogeneous multimodal data, automatically identify complex interactions among variables, and integrate preoperative characteristics, intraoperative haemodynamic parameters, and postoperative biochemical indicators (17–19). Previous studies (20, 21) have shown that machine learning models outperform conventional approaches in discriminative ability and calibration for predicting perioperative mortality, major cardiovascular events, and postoperative complications.

Nevertheless, machine learning-based prediction models specifically targeting postoperative cardiac arrest in elderly patients remain scarce, and dedicated risk stratification approaches integrating multidimensional clinical information across the perioperative period are still limited. Accordingly, this study aimed to systematically evaluate established machine learning algorithms for predicting postoperative IHCA in elderly patients undergoing non-cardiac surgery, externally validate the best-performing model in an independent cohort, and characterize the relative contribution of key perioperative predictors using model interpretability analyses. The model is intended to be used during the early postoperative period, based on clinical information available before the occurrence of IHCA, to identify elderly surgical patients at increased risk and facilitate targeted postoperative risk stratification. The intended users are surgeons, anesthesiologists, intensivists, and other clinicians involved in the perioperative and postoperative management of elderly surgical patients. Rather than replacing clinical judgment or prescribing a specific intervention, the model is intended to help clinicians distinguish patients who may benefit from more intensive and individualized postoperative monitoring and management from those at relatively lower risk, thereby supporting more targeted allocation of clinical attention and monitoring resources.

## Materials and methods

### Study population and data sources

This multicenter retrospective cohort study included elderly surgical patients from six tertiary hospitals in China, namely Wuxi People's Hospital Affiliated to Nanjing Medical University, Wuxi Second People's Hospital, Yixing People's Hospital, Tengzhou Central People's Hospital, Gaomi People's Hospital, and Tengzhou Hospital of Traditional Chinese Medicine. All patients undergoing surgery between January 2020 and January 2025 were screened for eligibility.

Patients were included if they met the following criteria: (1) age ≥65 years; (2) underwent non-cardiac surgical procedures during the study period, including general surgery, thoracic surgery, vascular surgery, orthopedics, neurosurgery, urology, gynecology, and otorhinolaryngology-head and neck surgery; representative procedures included but were not limited to radical gastrectomy, colorectal cancer resection, hepatectomy, lung cancer resection, esophagectomy, total hip or knee arthroplasty, spinal surgery, brain tumor resection, nephrectomy, cystectomy, prostate surgery, and gynecological oncologic surgery; (3) surgeries were performed by senior attending surgeons with independent operative qualifications, and patients were transferred postoperatively to a general ward, post-anesthesia care unit (PACU), or intensive care unit (ICU) for monitoring and management; and (4) complete perioperative clinical data were available, including demographic characteristics, comorbidities, preoperative laboratory tests, intraoperative records, and postoperative laboratory parameters.

Patients were excluded if they met any of the following criteria: (1) undergoing cardiac or major vascular surgery, including coronary artery bypass grafting, valve replacement, congenital heart disease repair, or procedures requiring cardiopulmonary bypass; (2) preoperative cardiac arrest or receipt of cardiopulmonary resuscitation (CPR) or extracorporeal membrane oxygenation (ECMO) before surgery; (3) abnormal preoperative cardiac biomarkers (troponin I/T, BNP, or D-dimer) within 48 h prior to surgery or known structural heart disease; (4) severe hepatic, renal, or other end-stage organ dysfunction; (5) multiple surgeries during the same hospitalization; and (6) incomplete perioperative clinical data.

This retrospective study was approved by the Ethics Committees of Wuxi People's Hospital Affiliated to Nanjing Medical University, Wuxi Second People's Hospital, Yixing People's Hospital, Tengzhou Central People's Hospital, Gaomi People's Hospital, and Tengzhou Hospital of Traditional Chinese Medicine. The study was conducted in accordance with the relevant ethical standards and approved under protocol numbers KY22086, Lunshen2022ke158, 2023-Y28 and IEC-AF/17−1.1.

### Clinical variables

Demographic characteristics, preoperative comorbidities, intraoperative variables, and early postoperative laboratory parameters were extracted from the electronic medical record system of each participating center. The collected variables included sex, age, body mass index (BMI), American Society of Anesthesiologists (ASA) classification, serum albumin (ALB), Nutritional Risk Screening 2002 (*N*RS2002) score, drinking history, smoking history, surgical history, anemia, hypothyroidism, hyperlipidemia, hypertension, diabetes mellitus, chronic obstructive pulmonary disease (COPD), tumor history, anesthesia type, surgical procedure, emergency surgery, operative duration, intraoperative blood loss, intraoperative hypotension, intraoperative tachycardia, intraoperative oxygen saturation (SpO₂), and intraoperative blood transfusion.

Postoperative laboratory variables included procalcitonin (PCT), C-reactive protein (CRP), serum amyloid A (SAA), neutrophil-to-lymphocyte ratio (*N*LR), serum creatinine (SCr), blood urea nitrogen (BUN), proteinuria, hematuria, B-type natriuretic peptide (BNP), cardiac troponin T (cTnT), cardiac troponin I (cTnI), lactate, D-dimer, and serum sodium, potassium, and calcium levels.

To ensure a clearly defined temporal relationship between predictor assessment and the outcome, the timing of acquisition of all clinical variables and outcome events was explicitly determined. For patients who did not experience IHCA, postoperative laboratory variables were defined as the first measurements obtained within 24 h after surgery. The same temporal definition was applied to patients who subsequently developed IHCA, provided that the corresponding laboratory measurements were obtained before the onset of IHCA. Patients who developed IHCA before the completion of the postoperative laboratory assessment were excluded from the analysis because the prespecified postoperative laboratory predictors would not have been available before the outcome occurred. This approach ensured that all predictor variables incorporated into the model were available before the occurrence of IHCA and minimized the potential for information leakage. Accordingly, the model was designed to use early postoperative information to identify patients at increased risk of IHCA during the subsequent 30-day postoperative period.

### Outcome definition and clinical variable definitions

The primary prediction target was the occurrence of postoperative IHCA within 30 days after the index surgical procedure. The prediction model incorporated preoperative characteristics, intraoperative variables, and prespecified early postoperative clinical and laboratory measurements that were available before the occurrence of IHCA. To preserve the temporal integrity of the prediction framework and avoid information leakage, any postoperative clinical characteristics or laboratory measurements recorded after the onset of IHCA were excluded from the predictor set. Thus, the model estimated the risk of IHCA during the 30-day postoperative period using only information available before the occurrence of the event. Postoperative IHCA was defined as the sudden cessation of effective cardiac mechanical activity occurring within 30 days after surgery, resulting in the absence of effective circulation and characterized by loss of consciousness, absence of a palpable central pulse, and apnea or agonal respiration. Electrocardiographic manifestations included ventricular fibrillation (VF), pulseless ventricular tachycardia (pVT), pulseless electrical activity (PEA), or asystole. A cardiac arrest event was considered confirmed when the patient required chest compressions, defibrillation, cardioversion, or advanced cardiovascular life support (ACLS). All suspected events were identified and verified using electronic medical records, anesthesia records, intensive care unit documentation, and resuscitation logs. Each event was independently reviewed by two investigators, with discrepancies adjudicated by a senior physician (22, 23).

Intraoperative tachycardia was defined as a sustained heart rate >100 beats per minute lasting for at least 5 min during the surgical procedure, or requiring pharmacological intervention for rate control (e.g., beta-blockers or antiarrhythmic agents), as recorded in the anesthesia monitoring system (24, 25).

Intraoperative hypotension was defined as a mean arterial pressure (MAP) < 65 mmHg lasting for at least 10 min, or any episode of sustained hypotension requiring vasopressor administration (such as norepinephrine, ephedrine, or phenylephrine) to maintain hemodynamic stability, according to intraoperative anesthesia records (26, 27).

Intraoperative oxygen saturation (SpO₂) was continuously monitored using pulse oximetry, with the sensor typically placed on the finger or toe. SpO₂ data were continuously recorded through the intraoperative anesthesia monitoring system at a sampling frequency of at least 1 Hz and were retrospectively extracted and reviewed by trained investigators. Intraoperative hypoxaemia was prespecified as an SpO₂ < 90% sustained for at least 5 consecutive minutes during the maintenance phase of anaesthesia. The threshold of SpO₂ < 90% was selected based on its established use as a clinically relevant operational definition of perioperative hypoxaemia. The 5-minute duration criterion was prespecified to distinguish sustained impairment of oxygenation from brief, transient desaturation associated with routine anaesthetic procedures or temporary physiological perturbations, such as airway suctioning, changes in patient positioning, or transient surgical stimulation. Importantly, preoxygenation before induction, transient changes in oxygenation during induction and tracheal intubation, routine adjustments in FiO₂ during maintenance anaesthesia, and planned increases in oxygen supplementation before extubation were considered components of standard anaesthetic management and were not classified as hypoxaemia unless accompanied by an SpO₂ < 90% sustained for at least 5 consecutive minutes during the maintenance phase. Accordingly, this definition was based on objectively measured impairment of oxygenation rather than on the use or escalation of respiratory support or other routine anaesthetic interventions (28–30).

### Missing data

Missing data were handled using a prespecified strategy based on the proportion of missing observations for each candidate predictor to minimize potential bias while retaining as much information as possible for model development. Before model construction, the proportion of missing observations was calculated for each candidate variable. Variables with <5% missingness were handled using complete-case analysis, as the relatively small proportion of missing observations was considered unlikely to substantially affect the estimates. Variables with 5%–15% missingness were addressed using multiple imputation. Five imputed datasets were generated (m = 5), with 10 iterative cycles performed for each imputation procedure. Multiple imputation was preferred over single-value imputation because it accounts for uncertainty associated with missing values and helps reduce potential selection bias and loss of statistical efficiency resulting from complete-case exclusion. Variables with >30% missingness were excluded from the candidate predictor pool because the high proportion of missing information was considered insufficiently reliable for subsequent feature selection and model development. This prespecified approach was applied consistently before model construction and was independent of the observed outcome.

### Statistical analysis and feature selection

Continuous variables were expressed as mea*n* ± standard deviation or median (interquartile range) and compared using Student's t-test or the Mann–Whitney U test, as appropriate. Categorical variables were presented as frequencies (percentages) and compared using the chi-square test or Fisher's exact test. Univariate and multivariable logistic regression analyses were performed to identify variables independently associated with IHCA. Variables with *P* < 0.05 in the univariate analyses were entered into the multivariable logistic regression model using a forward stepwise selection approach. Results were reported as odds ratios (ORs) with 95% confidence intervals (CIs).

All feature-selection procedures were performed exclusively within the internal training cohort to avoid information leakage. To improve the robustness of feature selection and reduce dependence on any single modeling approach, five representative machine learning algorithms were considered, including Extreme Gradient Boosting (XGBoost), Random Forest (RF), Multilayer Perceptron (MLP), Support Vector Machine (SVM), and K-Nearest Neighbor (KNN). These algorithms represent complementary modeling paradigms, including tree-based ensemble learning (XGBoost and RF), kernel-based learning (SVM), distance-based learning (KNN), and neural network–based learning (MLP). Their use allowed candidate predictors to be evaluated from different modeling perspectives and reduced reliance on the feature-selection characteristics of a single algorithm.

Feature-importance rankings were obtained separately for each machine learning algorithm using the corresponding model-specific feature-importance or attribution procedure. For XGBoost and RF, feature importance was derived from the contribution of individual variables to the fitted tree ensembles. For SVM, KNN, and MLP, feature importance was assessed using the corresponding model-based feature contribution approach implemented in the analytical framework. Variables were ranked according to their relative importance within each algorithm. The resulting rankings were then compared across the five algorithms to identify predictors that consistently demonstrated high predictive importance. Rather than relying on the ranking from a single machine learning algorithm, this cross-model comparison was used to identify relatively stable predictive signals across different modeling paradigms.

Finally, a consensus feature-selection strategy was applied by integrating the machine learning–based importance results with multivariable logistic regression. Variables that were independently associated with IHCA in the multivariable logistic regression analysis and simultaneously ranked among the top 10 features in the machine learning–based importance assessment were retained as candidate predictors. Thus, the final candidate predictor set represented the intersection of conventional statistical evidence and machine learning–based predictive importance. This strategy was intended to prioritize variables that demonstrated both independent associations with IHCA and consistent predictive relevance across multiple machine learning frameworks, thereby improving clinical interpretability and reducing potential redundancy. The selected predictors were subsequently used for development and comparison of the five predictive models.

### Model development and internal validation

Patients from Wuxi People's Hospital, Wuxi Second People's Hospital, and Yixing People's Hospital were assigned to the internal cohort, whereas patients from Tengzhou Central People's Hospital, Tengzhou Hospital of Traditional Chinese Medicine, and Gaomi People's Hospital constituted the external cohort. The internal cohort was first partitioned into training and internal validation sets using a reproducible stratified random sampling strategy. Specifically, patients were stratified according to the outcome status (i.e., postoperative IHCA) to minimize potential imbalance in the rare outcome caused by random variation, and were then randomly assigned to the training and validation sets at a 7:3 ratio using a fixed random seed of 42. The external cohort was kept completely independent and was not involved in cohort partitioning, feature selection, model development, or hyperparameter optimization.

Feature selection was subsequently performed exclusively within the training cohort. Candidate predictors were evaluated using the prespecified feature-selection strategy, and the final predictor set was determined without using any information from the internal validation or external cohorts. Based on the selected features, five predictive models, including XGBoost, RF, MLP, SVM, and KNN, were developed using the training cohort. Hyperparameter optimization was performed using cross-validation restricted to the training cohort. The internal validation cohort was used solely for independent evaluation of model performance, whereas the external cohort was subsequently used for independent external validation. Model performance was comprehensively evaluated using the area under the receiver operating characteristic curve (AUC), calibration curves, Brier scores, and decision curve analysis (DCA), assessing discrimination, calibration, and clinical utility, respectively.

To evaluate model robustness and reduce potential overfitting, 10-fold cross-validation was performed within the training cohort. Specifically, the training set was randomly divided into 10 approximately equal and mutually exclusive folds. In each iteration, nine folds were used for model training and the remaining fold was used for internal validation. This process was repeated 10 times so that each fold served once as the validation set. The mean AUC and accuracy across all folds were calculated to provide an unbiased estimate of model generalizability.

For the optimal model (XGBoost), additional internal diagnostic analyses were conducted to further characterize model behavior. Learning curves were generated by incrementally increasing the training sample size to evaluate whether model performance plateaued with increasing data, thereby assessing potential overfitting or underfitting. The Kolmogorov–Smirnov (K-S) statistic was calculated to quantify the maximum separation between the cumulative distributions of predicted probabilities for the positive and negative classes, providing a threshold-independent measure of discriminative ability. Furthermore, confusion matrices were constructed at the optimal probability cutoff to directly evaluate classification performance, including sensitivity, specificity, and misclassification patterns. Collectively, these analyses were performed to provide a comprehensive assessment of the model's learning dynamics, discriminative performance, and classification reliability within the internal dataset.

### External validation

Independent external validation of the final optimal model (XGBoost) was performed using an independent cohort from three hospitals that were not involved in the internal dataset, namely Tengzhou Central People's Hospital, Gaomi People's Hospital, and Tengzhou Hospital of Traditional Chinese Medicine. These external data were not used for feature selection, feature importance ranking, model selection, hyperparameter tuning, threshold determination, or any other model optimization procedure. All model development and optimization procedures were conducted exclusively using the internal training cohort. The external validation cohort was evaluated only after the final predictor set, model specification, hyperparameters, and decision threshold had been determined. Model performance in the external cohort was assessed in terms of discrimination (AUC), calibration (calibration curves with 1,000 bootstrap resamples and mean absolute error), and clinical utility (decision curve analysis).

### Comparison with conventional risk stratification tools

To provide a clinical benchmark for the discrimination of the machine learning–based models, two established perioperative risk stratification tools, the American Society of Anesthesiologists Physical Status classification (ASA-PS) and the Revised Cardiac Risk Index (RCRI), were evaluated as conventional comparators. ASA-PS was categorized according to its standard physical status classification, whereas the RCRI was calculated based on its established six clinical components, with patients classified into four risk categories according to the total score (0, 1, 2, and ≥3 points). Both scores were calculated using preoperative information only and were evaluated in the same internal validation cohort used for assessment of the predictive models. The discriminatory performance of ASA-PS and RCRI for postoperative IHCA was assessed using the area under the receiver operating characteristic curve (AUC) with 95% confidence intervals. The AUC of the best-performing machine learning model was subsequently compared with those of ASA-PS and RCRI in the same validation cohort to provide a standardized comparison with conventional perioperative risk stratification approaches.

### Model interpretability

To improve interpretability of the optimal XGBoost model, SHapley Additive exPlanations (SHAP) analysis was performed. Global feature importance and directional effects were visualized using SHAP summary plots, where color gradients (red indicating high values and blue indicating low values) illustrated the relationship between feature magnitude and predicted risk. Furthermore, SHAP decision plots were constructed for five representative elderly patients to demonstrate individualized prediction pathways, illustrating how combinations of clinical variables shifted baseline risk to final predicted probabilities.

### Statistical software

All statistical analyses were performed using R software (version 4.1.2; R Foundation for Statistical Computing, Vienna, Austria) and Python (version 3.9; Python Software Foundation, USA). Machine learning algorithms were implemented using the Scikit-learn and XGBoost libraries in Python. A two-sided *P*-value < 0.05 was considered statistically significant.

## Results

### Baseline characteristics of the internal and external cohorts

A total of 7,327 elderly surgical patients from six tertiary hospitals were included in this study. The internal cohort comprised 4,106 patients from Wuxi People's Hospital, Wuxi Second People's Hospital, and Yixing People's Hospital, while the external cohort included 3,221 patients from Tengzhou Central People's Hospital, Tengzhou Traditional Chinese Medicine Hospital, and Gaomi People's Hospital. Overall, 77 patients experienced postoperative IHCA, yielding an incidence of 1.05% (Figure 1).

**Figure 1:**
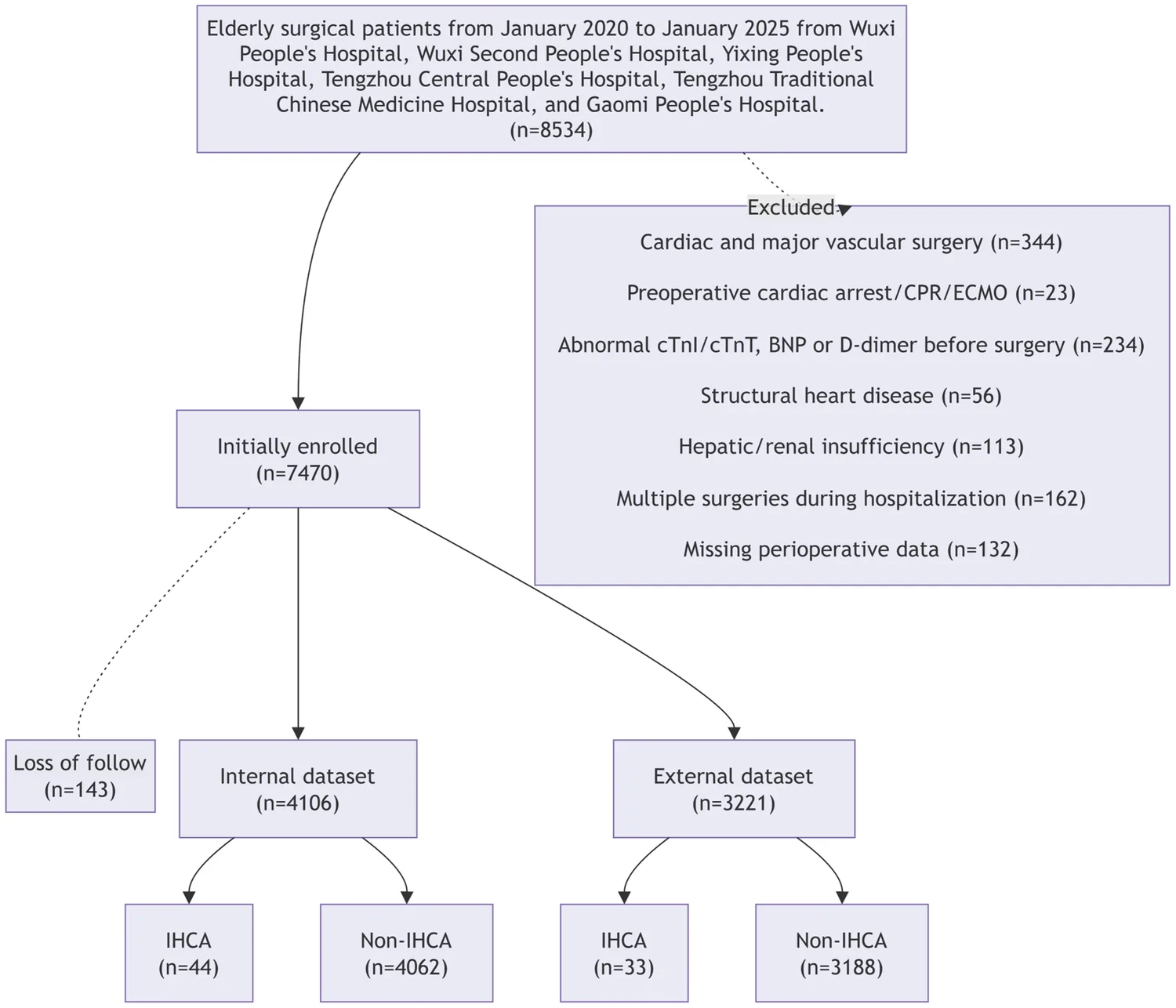
Study design nand patient selection flowchart.

Baseline characteristics are summarized in Table 1. The incidence of IHCA was comparable between the internal and external cohorts (1.07% vs. 1.03%, *P* = 0.845). However, significant inter-cohort differences were observed in several demographic, comorbidity, perioperative, and laboratory variables.

**Table 1:** Baseline characteristics of the internal and external cohorts. This table summarises the demographic characteristics, comorbidities, perioperative variables, and laboratory parameters of patients in the internal and external datasets.

Variables		All (*n* = 7,327)	Internal Dataset (*n* = 4,106)	External Dataset (*n* = 3,221)	*P*-value
Sex	Female	3,606 (49.215)	1,657 (40.356)	1,949 (60.509)	<0.001
	Male	3,721 (50.785)	2,449 (59.644)	1,272 (39.491)	
Age	＜80	5,139 (70.138)	2,885 (70.263)	2,254 (69.978)	0.792
	≥80	2,188 (29.862)	1,221 (29.737)	967 (30.022)	
BMI	＜25 kg/m^2^	4,691 (64.023)	2,453 (59.742)	2,238 (69.482)	<0.001
	≥25 kg/m^2^	2,636 (35.977)	1,653 (40.258)	983 (30.518)	
ASA	＜3	5,704 (77.849)	2,804 (68.290)	2,900 (90.034)	<0.001
	≥3	1,623 (22.151)	1,302 (31.710)	321 (9.966)	
ALB	≥30 g/L	5,741 (78.354)	2,876 (70.044)	2,865 (88.948)	<0.001
	＜30 g/L	1,586 (21.646)	1,230 (29.956)	356 (11.052)	
NRS2002 score	＜3	5,757 (78.572)	2,872 (69.946)	2,885 (89.568)	<0.001
	≥3	1,570 (21.428)	1,234 (30.054)	336 (10.432)	
Drinking history	No	5,937 (81.029)	3,275 (79.761)	2,662 (82.645)	0.002
	Yes	1,390 (18.971)	831 (20.239)	559 (17.355)	
Smoking history	No	6,013 (82.066)	3,305 (80.492)	2,708 (84.073)	<0.001
	Yes	1,314 (17.934)	801 (19.508)	513 (15.927)	
Surgical history	No	6,607 (90.173)	3,721 (90.623)	2,886 (89.600)	0.144
	Yes	720 (9.827)	385 (9.377)	335 (10.400)	
Anemia	No	7,079 (96.615)	4,076 (99.269)	3,003 (93.232)	<0.001
	Yes	248 (3.385)	30 (0.731)	218 (6.768)	
Hypothyroidism	No	6,906 (94.254)	3,945 (96.079)	2,961 (91.928)	<0.001
	Yes	421 (5.746)	161 (3.921)	260 (8.072)	
Hyperlipidemia	No	6,766 (92.343)	3,831 (93.302)	2,935 (91.121)	<0.001
	Yes	561 (7.657)	275 (6.698)	286 (8.879)	
Hypertension	No	6,646 (90.706)	3,750 (91.330)	2,896 (89.910)	0.038
	Yes	681 (9.294)	356 (8.670)	325 (10.090)	
Diabetes	No	6,761 (92.275)	3,840 (93.522)	2,921 (90.686)	<0.001
	Yes	566 (7.725)	266 (6.478)	300 (9.314)	
COPD	No	7,052 (96.247)	4,028 (98.100)	3,024 (93.884)	<0.001
	Yes	275 (3.753)	78 (1.900)	197 (6.116)	
Tumor	No	6,321 (86.270)	3,766 (91.719)	2,555 (79.323)	<0.001
	Yes	1,006 (13.730)	340 (8.281)	666 (20.677)	
Anesthesia type	General anesthesia	7,044 (96.138)	3,956 (96.347)	3,088 (95.871)	0.294
	Regional anesthesia	283 (3.862)	150 (3.653)	133 (4.129)	
Surgical procedure	Open surgery	6,097 (83.213)	3,493 (85.071)	2,604 (80.844)	<0.001
	Laparoscopic surgery	1,230 (16.787)	613 (14.929)	617 (19.156)	
Emergency surgery	No	5,827 (79.528)	3,261 (79.420)	2,566 (79.665)	0.797
	Yes	1,500 (20.472)	845 (20.580)	655 (20.335)	
Surgery time	＜270 min	4,739 (64.679)	2,448 (59.620)	2,291 (71.127)	<0.001
	≥270 min	2,588 (35.321)	1,658 (40.380)	930 (28.873)	
Intraoperative bleeding	＜100 mL	4,739 (64.679)	2,493 (60.716)	2,246 (69.730)	<0.001
	≥100 mL	2,588 (35.321)	1,613 (39.284)	975 (30.270)	
Intraoperative hypotension	No	6,605 (90.146)	3,652 (88.943)	2,953 (91.680)	<0.001
	Yes	722 (9.854)	454 (11.057)	268 (8.320)	
Intraoperative tachycardia	No	6,625 (90.419)	3,678 (89.576)	2,947 (91.493)	0.006
	Yes	702 (9.581)	428 (10.424)	274 (8.507)	
Intraoperative SpO_2_	≥90%	6,839 (93.340)	3,829 (93.254)	3,010 (93.449)	0.739
	＜90%	488 (6.660)	277 (6.746)	211 (6.551)	
Blood transfusion	No	6,812 (92.971)	3,752 (91.378)	3,060 (95.002)	<0.001
	Yes	515 (7.029)	354 (8.622)	161 (4.998)	
PCT level	＜0.05 ng/mL	6,488 (88.549)	3,797 (92.474)	2,691 (83.545)	<0.001
	≥0.05 ng/mL	839 (11.451)	309 (7.526)	530 (16.455)	
CRP level	＜10 mg/L	6,452 (88.058)	3,762 (91.622)	2,690 (83.514)	<0.001
	≥10 mg/L	875 (11.942)	344 (8.378)	531 (16.486)	
SAA level	＜10 mg/L	6,505 (88.781)	3,793 (92.377)	2,712 (84.197)	<0.001
	≥10 mg/L	822 (11.219)	313 (7.623)	509 (15.803)	
NLR	＜3	6,440 (87.894)	3,770 (91.817)	2,670 (82.894)	<0.001
	≥3	887 (12.106)	336 (8.183)	551 (17.106)	
SCr	＜1.2 mg/dL	6,240 (85.164)	3,693 (89.942)	2,547 (79.075)	<0.001
	≥1.2 mg/dL	1,087 (14.836)	413 (10.058)	674 (20.925)	
BUN	＜20 mg/dL	5,166 (70.506)	2,907 (70.799)	2,259 (70.133)	0.535
	≥20 mg/dL	2,161 (29.494)	1,199 (29.201)	962 (29.867)	
Proteinuria	No	6,165 (84.141)	3,261 (79.420)	2,904 (90.158)	<0.001
	Yes	1,162 (15.859)	845 (20.580)	317 (9.842)	
Hematuria	No	6,357 (86.761)	3,460 (84.267)	2,897 (89.941)	<0.001
	Yes	970 (13.239)	646 (15.733)	324 (10.059)	
BNP	＜400 pg/mL	5,413 (73.877)	2,844 (69.264)	2,569 (79.758)	<0.001
	≥400 pg/mL	1,914 (26.123)	1,262 (30.736)	652 (20.242)	
cTnT	＜0.03 ng/mL	6,167 (84.168)	3,587 (87.360)	2,580 (80.099)	<0.001
	≥0.03 ng/mL	1,160 (15.832)	519 (12.640)	641 (19.901)	
cTnl	＜0.04 ng/mL	6,118 (83.499)	3,566 (86.849)	2,552 (79.230)	<0.001
	≥0.04 ng/mL	1,209 (16.501)	540 (13.151)	669 (20.770)	
Lactate	＜2.0 mmol/L	5,980 (81.616)	3,287 (80.054)	2,693 (83.608)	<0.001
	≥2.0 mmol/L	1,347 (18.384)	819 (19.946)	528 (16.392)	
D-dimer	＜2.0 mg/L	5,837 (79.664)	3,290 (80.127)	2,547 (79.075)	0.267
	≥2.0 mg/L	1,490 (20.336)	816 (19.873)	674 (20.925)	
Sodium	Normal	4,899 (66.862)	2,456 (59.815)	2,443 (75.846)	<0.001
	Hyponatremia	1,165 (15.900)	839 (20.434)	326 (10.121)	
	Hypernatremia	1,263 (17.238)	811 (19.752)	452 (14.033)	
Potassium	Normal	5,876 (80.197)	3,376 (82.221)	2,500 (77.616)	<0.001
	Hypokalemia	733 (10.004)	427 (10.399)	306 (9.500)	
	Hyperkalemia	718 (9.799)	303 (7.379)	415 (12.884)	
Calcium	Normal	4,873 (66.507)	2,399 (58.427)	2,474 (76.808)	<0.001
	Hypocalcemia	1,134 (15.477)	805 (19.605)	329 (10.214)	
	Hypercalcemia	1,320 (18.016)	902 (21.968)	418 (12.977)	
IHCA	No	7,250 (98.949)	4,062 (98.928)	3,188 (98.975)	0.845
	Yes	77 (1.051)	44 (1.072)	33 (1.025)	

OR, odds ratio; CI, confidence interval; BMI, body mass index; IHCA, in-hospital cardiac arrest; D-dimer, D-dimer; cTnI, cardiac troponin I; cTnT, cardiac troponin T; BNP, B-type natriuretic peptide; BUN, blood urea nitrogen; SCr, serum creatinine; NLR, neutrophil-to-lymphocyte ratio; PCT, procalcitonin; CRP, C-reactive protein; SAA, serum amyloid A; SpO2, peripheral oxygen saturation; COPD, chronic obstructive pulmonary disease; NRS2,002 score, Nutritional Risk Screening 2,002 score; ALB, albumin; ASA, American Society of Anesthesiologists.

Compared with the internal cohort, the external cohort had higher proportions of female patients, anemia, hypothyroidism, hyperlipidemia, diabetes, chronic obstructive pulmonary disease, and tumor history, as well as higher levels of postoperative inflammatory markers, including PCT, CRP, SAA, and NLR (all *P* < 0.001). In contrast, the internal cohort showed higher rates of obesity (BMI ≥25 kg/m^2^), ASA class ≥3, hypoalbuminemia, nutritional risk (NRS2002 ≥ 3), prolonged operative duration, intraoperative bleeding, hypotension, tachycardia, transfusion, proteinuria, hematuria, elevated BNP, elevated cardiac troponin, and electrolyte disturbances (all *P* < 0.001).

No significant differences were observed in age distribution, surgical history, anesthesia type, emergency surgery status, intraoperative hypoxemia, blood urea nitrogen, D-dimer levels, or IHCA incidence (all *P* > 0.05).

The internal and external cohorts differed significantly in several baseline demographic, clinical, laboratory, and perioperative characteristics, indicating substantial heterogeneity between the development and external validation populations. The original dataset utilized in this study is provided in Supplementary Table S1.

### Identification of risk factors for postoperative IHCA

To identify potential predictors of postoperative IHCA in elderly surgical patients, univariate logistic regression analyses were initially performed on all candidate demographic, perioperative, and laboratory variables. The results are summarized in Table 2.

**Table 2:** Univariate and multivariate logistic regression analyses of risk factors for postoperative IHCA.

Variables		Univariate analysis		Multivariate analysis	
		OR, 95%CI	*P*-value	OR, 95%CI	*P*-value
Sex	Female	Reference		Reference	
	Male	0.464, [0.254, 0.850]	0.013	0.504, [0.134, 1.809]	0.293
Age	＜80	Reference		Reference	
	≥80	3.815, [2.072, 7.025]	<0.001	8.158, [2.245, 36.613]	0.003
BMI	＜25 kg/m^2^	Reference			
	≥25 kg/m^2^	1.028, [0.562, 1.880]	0.929		
ASA	＜3	Reference			
	≥3	0.715, [0.360, 1.420]	0.338		
ALB	≥30 g/L	Reference			
	＜30 g/L	0.876, [0.449, 1.706]	0.696		
NRS2002 score	＜3	Reference			
	≥3	1.472, [0.799, 2.710]	0.215		
Drinking history	No	Reference			
	Yes	0.743, [0.330, 1.674]	0.474		
Smoking history	No	Reference			
	Yes	1.556, [0.798, 3.034]	0.195		
Surgical history	No	Reference			
	Yes	1.242, [0.487, 3.171]	0.65		
Anemia	No	Reference			
	Yes	3.234, [0.431, 24.283]	0.254		
Hypothyroidism	No	Reference			
	Yes	1.169, [0.280, 4.872]	0.83		
Hyperlipidemia	No	Reference			
	Yes	1.399, [0.497, 3.939]	0.525		
Hypertension	No	Reference			
	Yes	0.499, [0.120, 2.069]	0.338		
Diabetes	No	Reference			
	Yes	0.333, [0.046, 2.429]	0.278		
COPD	No	Reference			
	Yes	1.204, [0.164, 8.852]	0.856		
Tumor	No	Reference			
	Yes	0.809, [0.249, 2.626]	0.724		
Anesthesia type	General anesthesia	Reference			
	Regional anesthesia	2.682, [0.947, 7.596]	0.063		
Surgical procedure	Open surgery	Reference			
	Laparoscopic surgery	1.270, [0.587, 2.745]	0.544		
Emergency surgery	No	Reference			
	Yes	0.607, [0.256, 1.440]	0.257		
Surgery time	＜270 min	Reference			
	≥270 min	0.686, [0.363, 1.298]	0.247		
Intraoperative bleeding	＜100 mL	Reference			
	≥100 mL	0.719, [0.380, 1.360]	0.31		
Intraoperative hypotension	No	Reference		Reference	
	Yes	13.520, [7.309, 25.010]	<0.001	16.876, [4.849, 71.564]	<0.001
Intraoperative tachycardia	No	Reference		Reference	
	Yes	22.014, [11.425, 42.416]	<0.001	41.482, [11.487, 197.585]	<0.001
Intraoperative SpO_2_	≥90%	Reference		Reference	
	＜90%	12.338, [6.726, 22.632]	<0.001	23.593, [6.535, 104.772]	<0.001
Blood transfusion	No	Reference		Reference	
	Yes	13.571, [7.418, 24.827]	<0.001	34.579, [9.383, 164.820]	<0.001
PCT level	＜0.05 ng/mL	Reference			
	≥0.05 ng/mL	1.232, [0.438, 3.466]	0.693		
CRP level	＜10 mg/L	Reference			
	≥10 mg/L	1.740, [0.730, 4.145]	0.211		
SAA level	＜10 mg/L	Reference			
	≥10 mg/L	0.886, [0.273, 2.876]	0.84		
NLR	＜3	Reference		Reference	
	≥3	9.879, [5.398, 18.080]	<0.001	23.711, [5.894, 119.917]	<0.001
SCr	＜1.2 mg/dL	Reference			
	≥1.2 mg/dL	0.893, [0.318, 2.509]	0.83		
BUN	＜20 mg/dL	Reference			
	≥20 mg/dL	1.391, [0.750, 2.580]	0.296		
Proteinuria	No	Reference			
	Yes	0.856, [0.397, 1.849]	0.693		
Hematuria	No	Reference			
	Yes	0.684, [0.269, 1.743]	0.426		
BNP	＜400 pg/mL	Reference		Reference	
	≥400 pg/mL	4.011, [2.162, 7.439]	<0.001	4.649, [1.342, 18.339]	0.019
cTnT	＜0.03 ng/mL	Reference		Reference	
	≥0.03 ng/mL	2.629, [1.346, 5.138]	0.005	4.492, [1.066, 19.466]	0.04
cTnl	＜0.04 ng/mL	Reference		Reference	
	≥0.04 ng/mL	3.485, [1.856, 6.543]	<0.001	6.025, [1.342, 30.130]	0.021
Lactate	＜2.0 mmol/L	Reference		Reference	
	≥2.0 mmol/L	7.237, [3.896, 13.441]	<0.001	9.334, [2.726, 36.843]	0.001
D-dimer	＜2.0 mg/L	Reference		Reference	
	≥2.0 mg/L	8.931, [4.713, 16.923]	<0.001	22.771, [6.186, 110.067]	<0.001
Sodium	Normal	Reference			
	Hyponatremia	1.010, [0.490, 2.080]	0.979		
	Hypernatremia	0.519, [0.200, 1.346]	0.177		
Potassium	Normal	Reference		Reference	
	Hypokalemia	7.036, [3.325, 14.890]	<0.001	14.734, [3.219, 78.711]	0.001
	Hyperkalemia	12.492, [6.113, 25.526]	<0.001	17.082, [4.167, 81.734]	<0.001
Calcium	Normal	Reference			
	Hypocalcemia	0.793, [0.362, 1.736]	0.561		
	Hypercalcemia	0.529, [0.219, 1.275]	0.156		

OR, odds ratio; CI, confidence interval; BMI, body mass index; IHCA, in-hospital cardiac arrest; D-dimer, D-dimer; cTnI, cardiac troponin I; cTnT, cardiac troponin T; BNP, B-type natriuretic peptide; BUN, blood urea nitrogen; SCr, serum creatinine; NLR, neutrophil-to-lymphocyte ratio; PCT, procalcitonin; CRP, C-reactive protein; SAA, serum amyloid A; SpO2, peripheral oxygen saturation; COPD, chronic obstructive pulmonary disease; NRS2,002 score, Nutritional Risk Screening 2,002 score; ALB, albumin; ASA, American Society of Anesthesiologists.

Univariate analysis demonstrated that advanced age (≥80 years), intraoperative hypotension, intraoperative tachycardia, intraoperative hypoxemia (SpO₂ < 90%), blood transfusion, elevated neutrophil-to-lymphocyte ratio (NLR ≥3), elevated B-type natriuretic peptide (BNP ≥400 pg/mL), elevated cardiac troponin T (cTnT ≥0.03 ng/mL), elevated cardiac troponin I (cTnI ≥0.04 ng/mL), elevated lactate (≥2.0 mmol/L), elevated D-dimer (≥2.0 mg/L), hypokalemia, and hyperkalemia were significantly associated with an increased risk of postoperative IHCA (all *P* < 0.05). Male sex was associated with a lower incidence of IHCA in univariate analysis (OR = 0.464, 95% CI: 0.254–0.850, *P* = 0.013). In contrast, most baseline comorbidities, nutritional indicators, inflammatory biomarkers, and operative characteristics did not demonstrate significant associations with IHCA.

Variables with statistical significance in the univariate analysis were subsequently entered into a multivariable logistic regression model to identify independent predictors of postoperative IHCA. As shown in Table 2, advanced age remained an independent risk factor, with patients aged ≥80 years demonstrating an approximately eight-fold increased risk of IHCA compared with younger elderly patients (OR = 8.158, 95% CI: 2.245–36.613, *P* = 0.003).

Several intraoperative adverse events emerged as the strongest independent predictors. Intraoperative tachycardia was associated with a more than forty-fold increase in risk (OR = 41.482, 95% CI: 11.487–197.585, *P* < 0.001), representing the most influential predictor identified in the regression model. Blood transfusion (OR = 34.579, 95% CI: 9.383–164.820, *P* < 0.001), intraoperative hypoxemia (OR = 23.593, 95% CI: 6.535–104.772, *P* < 0.001), and intraoperative hypotension (OR = 16.876, 95% CI: 4.849–71.564, *P* < 0.001) were also independently associated with markedly increased risks of postoperative cardiac arrest.

Regarding laboratory biomarkers, NLR ≥3 emerged as a strong independent predictor (OR = 23.711, 95% CI: 5.894–119.917, *P* < 0.001). Similarly, D-dimer ≥2.0 mg/L was associated with a substantially elevated risk of IHCA (OR = 22.771, 95% CI: 6.186–110.067, *P* < 0.001), suggesting a close relationship between coagulation dysfunction and adverse cardiovascular outcomes.

Markers reflecting cardiac injury and dysfunction also remained independently associated with postoperative cardiac arrest. Elevated BNP (OR = 4.649, 95% CI: 1.342–18.339, *P* = 0.019), cTnT (OR = 4.492, 95% CI: 1.066–19.466, *P* = 0.040), and cTnI (OR = 6.025, 95% CI: 1.342–30.130, *P* = 0.021) were all identified as independent risk factors. Furthermore, elevated lactate levels (OR = 9.334, 95% CI: 2.726–36.843, *P* = 0.001) indicated that tissue hypoperfusion and metabolic stress were strongly associated with subsequent cardiac arrest events.

Electrolyte disturbances were also important contributors. Compared with patients with normal serum potassium levels, hypokalemia was associated with a 14.7-fold increased risk of IHCA (OR = 14.734, 95% CI: 3.219–78.711, *P* = 0.001), while hyperkalemia was associated with a 17.1-fold increased risk (OR = 17.082, 95% CI: 4.167–81.734, *P* < 0.001). Notably, although male sex was significant in the univariate analysis, it lost statistical significance after adjustment for confounding factors (OR = 0.504, 95% CI: 0.134–1.809, *P* = 0.293).

### Feature selection using machine learning algorithms

To identify the most informative predictors and reduce feature redundancy, five machine learning algorithms, including XGBoost, RF, MLP, SVM, and KNN, were employed for feature selection. The feature importance rankings generated by the five algorithms are presented in Figures 2A–E.

**Figure 2:**
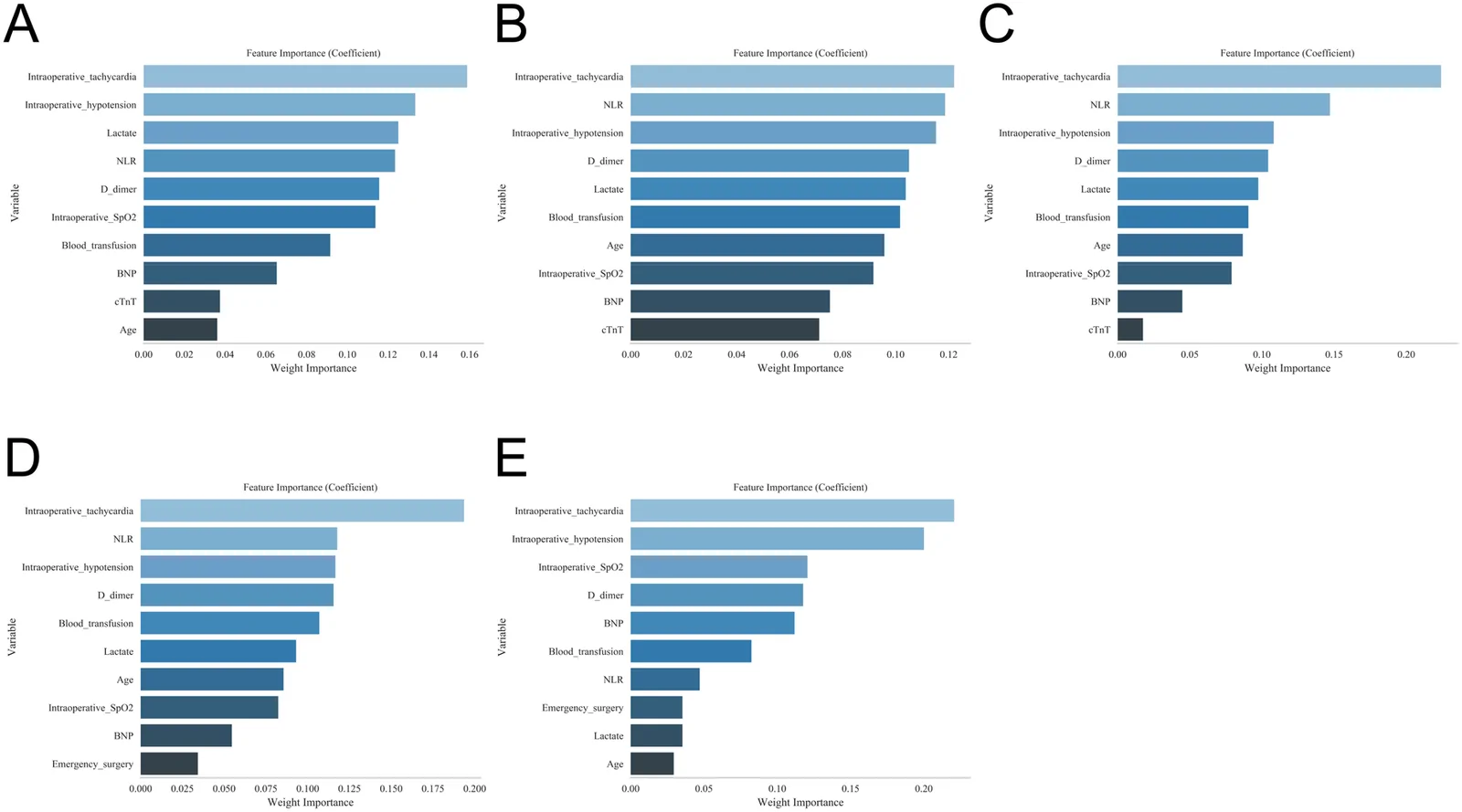
Feature selection based on machine learning algorithms. This figure presents the ranking of clinical feature importance derived from five machine learning models: **(A)** XGBoost, **(B)** random forest, **(C)** multilayer perceptron, **(D)** support vector machine, and **(E)** k-nearest neighbours.

Hyperparameter optimization was performed using grid search with stratified 10-fold cross-validation within the training cohort. For XGBoost, the candidate values were 100, 200, and 300 for n_estimators; 3, 4, and 5 for max_depth; 0.01, 0.05, and 0.10 for learning_rate; 1, 3, and 5 for min_child_weight; 0.8, 0.9, and 1.0 for both subsample and colsample_bytree; 0, 0.01, and 0.1 for reg_alpha; and 1, 5, and 10 for reg_lambda. The optimal XGBoost configuration was n_estimators = 200, max_depth = 3, learning_rate = 0.05, min_child_weight = 1, subsample = 0.9, colsample_bytree = 0.9, reg_alpha = 0.01, and reg_lambda = 1. For RF, n_estimators was searched over 100, 200, and 300; max_depth over 3, 5, 7, and None; min_samples_split over 2, 5, and 10; min_samples_leaf over 1, 2, and 4; and max_features over sqrt, log2, and 0.5. The optimal configuration was n_estimators = 200, max_depth = 5, min_samples_split = 2, min_samples_leaf = 1, and max_features = sqrt. For MLP, the candidate hidden-layer architectures were (32), (64,), (32, 16), and (64,32), with activation functions of ReLU or tanh, alpha values of 0.0001, 0.001, and 0.01, learning_rate strategies of constant or adaptive, learning_rate_init values of 0.001 and 0.01, and max_iter values of 500 and 1000. The optimal configuration was hidden_layer_sizes = (32,16), ReLU activation, alpha = 0.001, adaptive learning rate, learning_rate_init = 0.001, and max_ite*r* = 1000. For SVM, an RBF kernel was used, with C values of 0.1, 1, 10, and 100 and gamma values of 0.001, 0.01, 0.1, 1, scale, and auto. The optimal configuration was C = 10 and gamma = 0.01. For KNN, n_neighbors values of 3, 5, 7, 9, and 11, weights of uniform and distance, and Euclidean, Manhattan, and Minkowski distance metrics were evaluated. The optimal configuration was n_neighbors = 5, distance-weighted voting, and the Euclidean distance metric. The code used in this study has been made publicly available through Nutstore and can be accessed at the following link: https://www.jianguoyun.com/p/DWh9chMQl-GKDBjEj-sFIAA.

The five machine learning algorithms consistently identified a subset of clinically relevant variables associated with postoperative IHCA, including age, intraoperative hypotension, intraoperative tachycardia, intraoperative hypoxemia (SpO₂ < 90%), blood transfusion, NLR, BNP, lactate, and D-dimer. Although slight differences existed among the individual algorithms, substantial overlap was observed in the selected features, suggesting stable predictive contributions across different modeling approaches.

To improve both robustness and clinical interpretability, the machine learning-based feature selection results were further integrated with the findings from univariate and multivariable logistic regression analyses. Variables consistently identified by both statistical analyses and machine learning algorithms were retained for subsequent model development.

Ultimately, nine predictors were selected as the final candidate variables: age, intraoperative hypotension, intraoperative tachycardia, intraoperative hypoxemia (SpO₂ < 90%), blood transfusion, NLR, BNP, lactate, and D-dimer.

### Model development and comparison

Among the five predictive models developed using the selected predictors, including XGBoost, RF, MLP, SVM, and KNN, the internal cohort was randomly partitioned into training and validation sets at a 7:3 ratio using stratified sampling. XGBoost demonstrated the highest discriminative performance in the prespecified split-sample analysis, with an AUC of 0.967 (95% CI: 0.932–0.998) in the training set and 0.960 (95% CI: 0.915–0.999) in the held-out internal validation set, respectively. The other models showed comparatively lower AUCs in the internal validation set, ranging from 0.585 to 0.759 (Figures 3A,B; Table 3).

**Figure 3:**
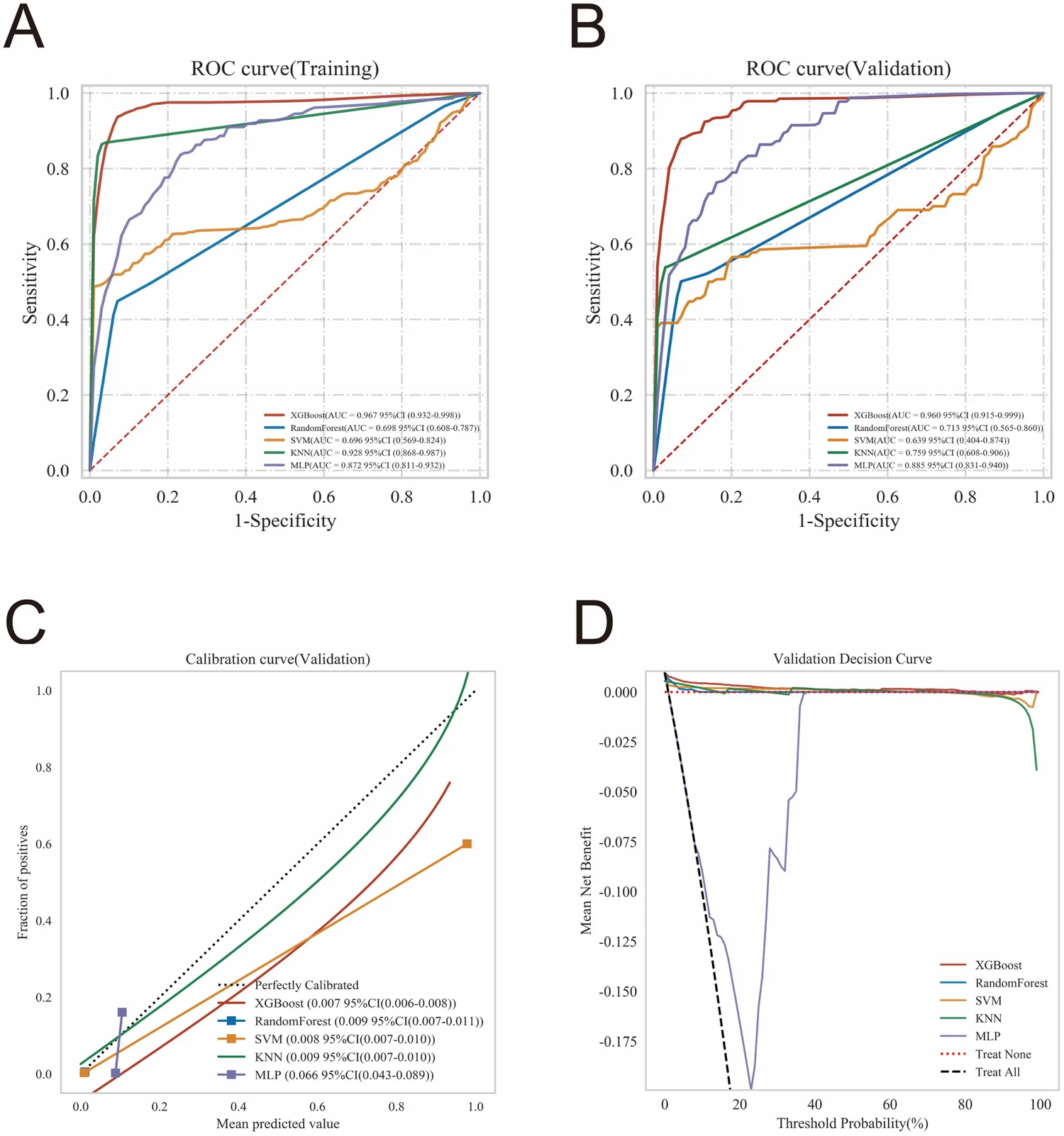
Performance comparison of machine learning models in the internal cohort. **(A,B)** Receiver operating characteristic (ROC) curves in the training and validation sets. **(C)** Calibration curves comparing predicted probabilities with observed outcomes. **(D)** Decision curve analysis (DCA) evaluating clinical net benefit across different threshold probabilities.

**Table 3:** Performance comparison of five machine learning models. The predictive performance of five machine learning models (XGBoost, random forest, multilayer perceptron, support vector machine, and k-nearest neighbours) was evaluated. Model performance was assessed using the AUC, accuracy, and other relevant metrics.

Models	Datasets	AUC (95%CI)	Accuracy (95%CI)	Sensitivity (95%CI)	Specificity (95%CI)	F1-score (95%CI)	Kappa (95%CI)
KNN	Training	0.928 (0.868–0.987)	0.914 (0.902–0.926)	0.761 (0.708–0.814)	0.922 (0.911–0.933)	0.396 (0.352–0.440)	0.351 (0.307–0.395)
	Validation	0.759 (0.608–0.906)	0.886 (0.865–0.907)	0.521 (0.438–0.604)	0.903 (0.886–0.920)	0.248 (0.194–0.302)	0.214 (0.161–0.267)
XGBoost	Training	0.967 (0.932–0.998)	0.934 (0.924–0.944)	0.891 (0.858–0.924)	0.936 (0.927–0.945)	0.542 (0.489–0.595)	0.506 (0.453–0.559)
	Validation	0.960 (0.915–0.999)	0.928 (0.916–0.940)	0.857 (0.789–0.926)	0.932 (0.920–0.944)	0.488 (0.417–0.559)	0.452 (0.381–0.523)
RF	Training	0.698 (0.608–0.787)	0.882 (0.869–0.895)	0.442 (0.389–0.495)	0.904 (0.895–0.913)	0.201 (0.171–0.231)	0.167 (0.137–0.197)
	Validation	0.713 (0.565–0.860)	0.878 (0.861–0.895)	0.491 (0.392–0.590)	0.897 (0.885–0.909)	0.214 (0.170–0.258)	0.181 (0.137–0.225)
SVM	Training	0.696 (0.569–0.824)	0.876 (0.852–0.900)	0.538 (0.460–0.616)	0.891 (0.870–0.912)	0.253 (0.203–0.303)	0.217 (0.167–0.267)
	Validation	0.639 (0.404–0.874)	0.869 (0.842–0.896)	0.401 (0.297–0.505)	0.889 (0.867–0.911)	0.181 (0.132–0.230)	0.145 (0.096–0.194)
MLP	Training	0.872 (0.811–0.932)	0.842 (0.816–0.868)	0.824 (0.779–0.869)	0.843 (0.818–0.868)	0.298 (0.255–0.341)	0.262 (0.219–0.305)
	Validation	0.885 (0.831–0.940)	0.835 (0.806–0.864)	0.871 (0.820–0.922)	0.833 (0.804–0.862)	0.311 (0.262–0.360)	0.275 (0.226–0.324)

CI, confidence interval; KNN, k-nearest neighbor; XGBoost, extreme gradient boosting; RF, random forest; SVM, support vector machine; MLP, multilayer perceptron; AUC, area under the curve.

In terms of calibration performance, calibration curves showed generally good agreement between predicted and observed probabilities for the XGBoost, RF, SVM, and KNN models, whereas the MLP model demonstrated a more pronounced calibration deviation. The corresponding Brier scores were 0.007 (95% CI: 0.006–0.008), 0.009 (95% CI: 0.007–0.011), 0.008 (95% CI: 0.007–0.010), 0.009 (95% CI: 0.007–0.010), and 0.066 (95% CI: 0.043–0.089) for XGBoost, RF, SVM, KNN, and MLP, respectively (Figure 3C). Given the low incidence of IHCA, these absolute Brier scores were interpreted cautiously and in conjunction with calibration curves and other complementary measures of model performance (Figure 3C).

Furthermore, DCA revealed the potential clinical utility of each model. Across most threshold probability ranges, the XGBoost, RF, SVM, and KNN models provided greater net benefit than either the “treat-all' or “treat-none' strategies. Notably, the XGBoost model consistently achieved the highest net benefit across a wide range of thresholds, underscoring its superior clinical applicability. By contrast, the MLP model demonstrated inferior net benefit across multiple threshold ranges, occasionally falling below the default strategies, thereby indicating limited clinical utility (Figure 3D). Collectively, XGBoost emerged as the optimal model, demonstrating the best overall performance in discrimination, calibration, and clinical net benefit, and thus the greatest translational potential.

To further assess model stability during development, stratified 10-fold cross-validation was performed within the training cohort. XGBoost achieved a mean cross-validation AUC of 0.8905 ± 0.0693 across the 10 validation folds. Following completion of model development and hyperparameter optimization, the final XGBoost model achieved an AUC of 0.9387 and an accuracy of 0.8807 in the subsequent model evaluation. The corresponding cross-validation and final evaluation results for SVM, MLP, RF, and KNN were 0.8871 ± 0.0110 and 0.8824, 0.8850 ± 0.0194 and 0.8839, 0.8425 ± 0.0365 and 0.8961, and 0.8220 ± 0.0240 and 0.7653, respectively. Overall, XGBoost demonstrated consistently strong discrimination across the different model-development procedures and was selected as the final predictive model (Figures 4A–C).

**Figure 4:**
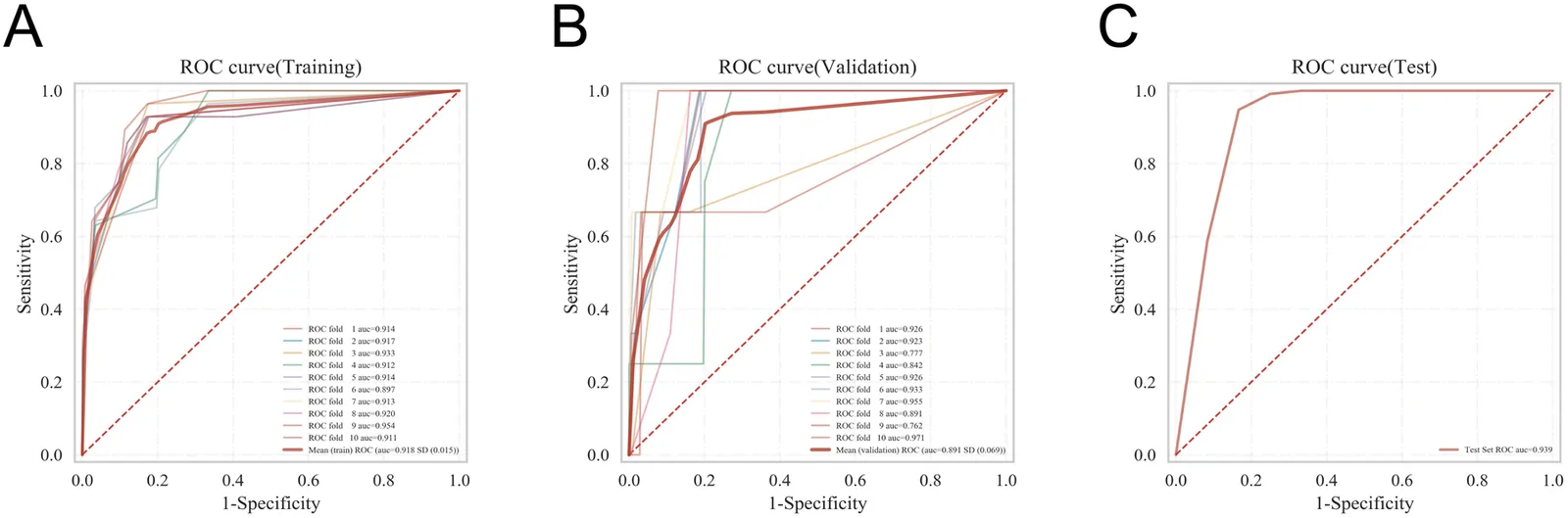
Internal validation and cross-validation performance of the models. Model stability and generalisability were assessed using 10-fold cross-validation. **(A)** AUC in the training set. **(B)** AUC in the validation set. **(C)** AUC in the test set.

To contextualize the discriminatory performance of our machine learning–based model, we compared it with two established perioperative risk stratification tools: the American Society of Anesthesiologists physical status classification (ASA-PS) and the Revised Cardiac Risk Index (RCRI). Both scoring systems were calculated using the corresponding preoperative variables and evaluated in the same internal validation cohort used for assessment of our predictive models (*n* = 1,467). The ASA-PS demonstrated poor discrimination for postoperative IHCA, with an AUC of 0.534 (95% CI, 0.469–0.594), whereas the RCRI showed moderate discriminatory ability, with an AUC of 0.751 (95% CI, 0.677–0.826) (Supplementary Figure S1A-B). In comparison, the XGBoost-based model achieved a higher AUC in the same validation cohort. These findings indicate that the machine learning–based model demonstrated better discriminatory performance for postoperative IHCA than the conventional perioperative risk stratification tools evaluated in this study.

To further interrogate the learning dynamics, discriminative capacity, and classification performance of the XGBoost model, a series of additional analyses were conducted. The learning curve (Figure 5A) showed a progressive increase in AUC with expanding training sample size in both training and validation sets, with the gap between the two curves gradually narrowing and converging. This pattern indicates adequate learning of informative features without evidence of overfitting or underfitting. The KS test (Figure 5B) further quantified the maximum cumulative separation between positive and negative classes, yielding a KS statistic of 0.931 (optimal cut-off threshold: 0.095), substantially exceeding commonly accepted benchmarks (>0.50) for strong discriminatory performance, thereby confirming excellent class separability. Confusion matrix analysis demonstrated high classification accuracy: in the training set (Figure 5C), the model correctly identified the majority of negative cases, while achieving 28 true positives with only 3 false negatives; in the validation set (Figure 5D), it correctly identified 12 positive cases with only 1 missed case, maintaining robust sensitivity despite class imbalance.

**Figure 5:**
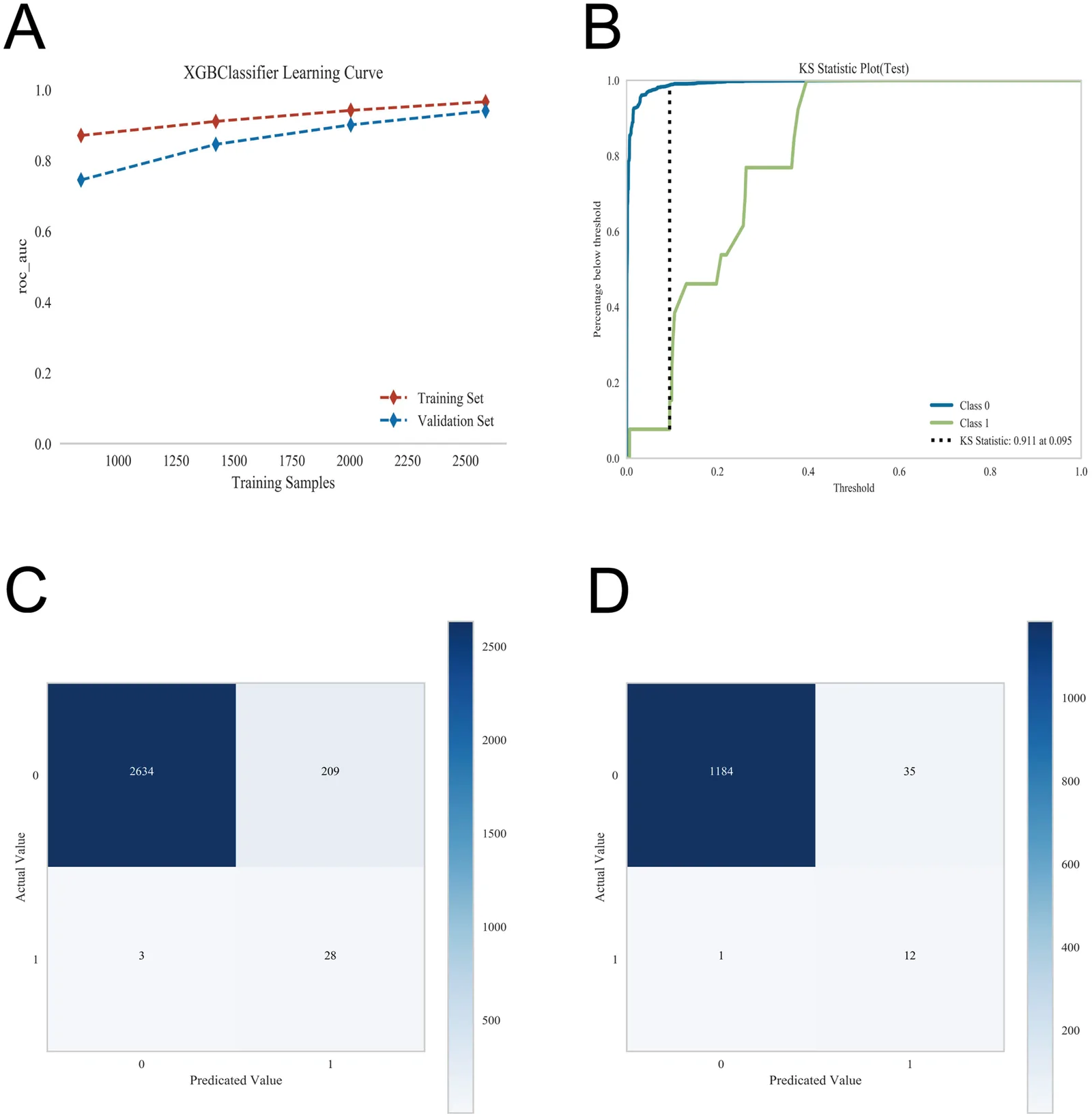
Internal diagnostic evaluation of the XGBoost model. **(A)** Learning curve illustrating performance trends as training sample size increases. **(B)** Kolmogorov–Smirnov (K–S) curve demonstrating class separability between positive and negative outcomes. **(C)** Confusion matrix for the training set. **(D)** Confusion matrix for the validation set.

To further validate the generalisability and robustness of the optimal XGBoost model, an independent external dataset was used for comprehensive evaluation. ROC analysis (Figure 6A) demonstrated excellent discriminative performance, with an AUC of 0.971 (95% CI: 0.950–0.991), indicating outstanding ability to distinguish outcome events. Calibration assessment (Figure 6B) showed strong agreement between predicted and observed probabilities, with both apparent and bias-corrected curves closely aligned with the ideal diagonal. Bootstrap resampling (1,000 iterations) yielded a mean absolute error of only 0.002, further confirming excellent calibration without evidence of overfitting or systematic bias. Decision curve analysis (Figure 6C) demonstrated that across a wide range of threshold probabilities (approximately 0%–70%), the XGBoost model consistently provided greater net benefit than both “treat-all' and “treat-none' strategies, indicating substantial clinical utility. Collectively, these external validation results confirm that the XGBoost model not only performs robustly in internal validation but also maintains excellent discrimination, calibration, and clinical applicability in an independent cohort, underscoring its strong external validity and translational potential.

**Figure 6:**
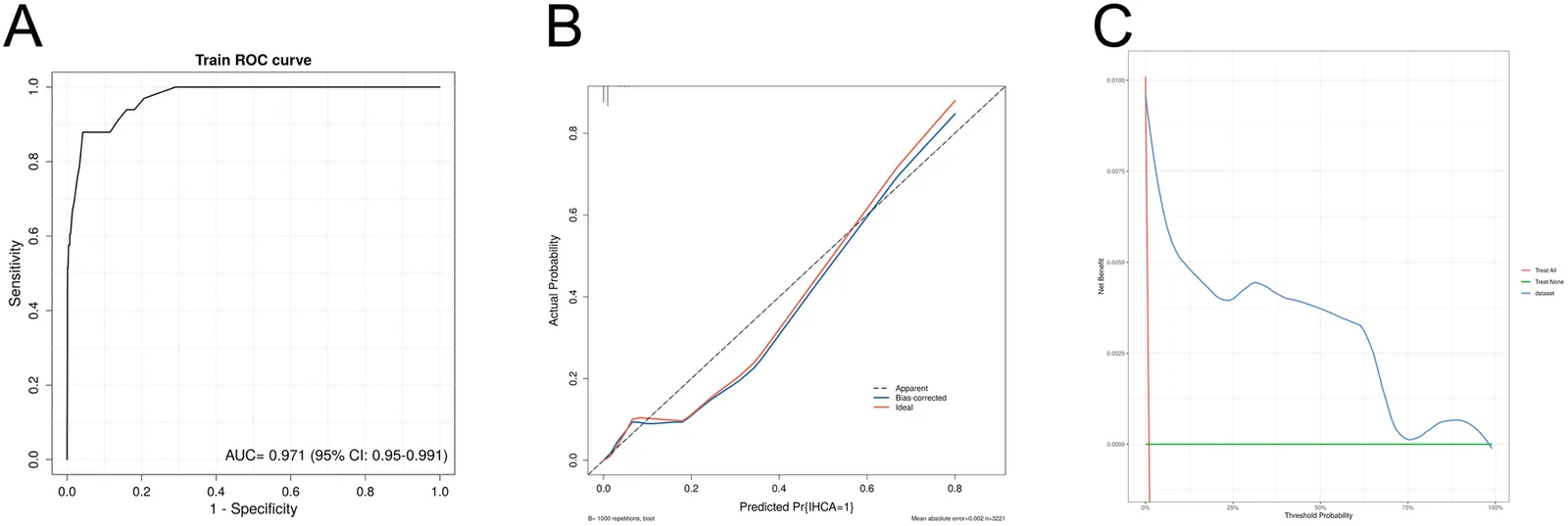
External validation of the XGBoost model. **(A)** ROC curve demonstrating discriminative performance in the external dataset. **(B)** Calibration plot with bootstrap resampling (1,000 iterations). **(C)** Decision curve analysis.

### Model interpretability: SHAP analysis

To further elucidate the internal decision-making mechanisms of the XGBoost model and enhance the interpretability of this inherently black-box framework, SHAP analysis was performed to quantify both global and local feature contributions. The SHAP summary plot (Figure 7) provides an intuitive visualization of the magnitude and direction of each clinical feature's contribution to the risk of postoperative cardiac arrest. Features were ranked according to their mean absolute SHAP values, thereby reflecting global importance. Among all variables, intraoperative tachycardia, lactate, and D-dimer emerged as the top three most influential predictors, indicating that these parameters constitute the principal driving forces of the model.

**Figure 7:**
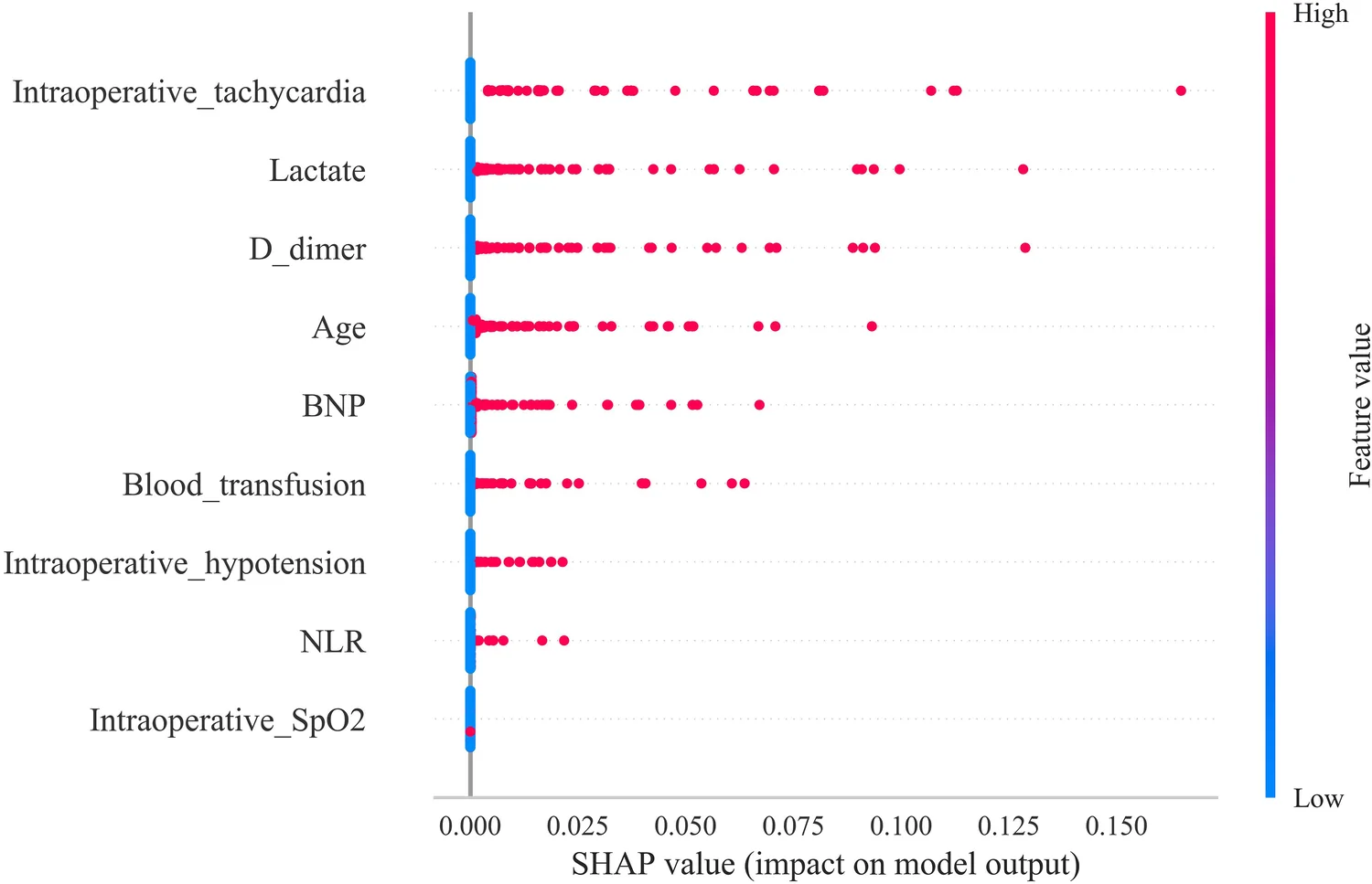
Global interpretability of the XGBoost model based on SHAP analysis. This figure illustrates feature importance ranking and directionality of effects. Higher feature values (red) and lower feature values (blue) indicate their contribution to increased or decreased risk of postoperative IHCA.

In terms of effect directionality, the colour gradient (with red indicating higher feature values and blue indicating lower values) further delineated the relationship between each variable and predicted risk. Notably, high-value observations for most key features were predominantly distributed on the positive SHAP axis (SHAP > 0), indicating an increased predicted probability of cardiac arrest. Specifically, intraoperative tachycardia, elevated lactate levels, increased D-dimer, advanced age, elevated BNP, intraoperative blood transfusion, intraoperative hypotension, hypoxaemia, and an elevated NLR were all strongly associated with a heightened risk of postoperative cardiac arrest.

Beyond global interpretation, SHAP decision plots were further constructed to illustrate individualized risk evolution in five elderly patients with heterogeneous clinical profiles. As shown in Figures 8A–8E, corresponding to patients 1–5, the baseline prediction values were uniformly low (0.00–0.01); however, due to substantial inter-individual clinical heterogeneity, the final predicted risk scores varied considerably [f(x) ranging from 0.01 to 0.25].

**Figure 8:**
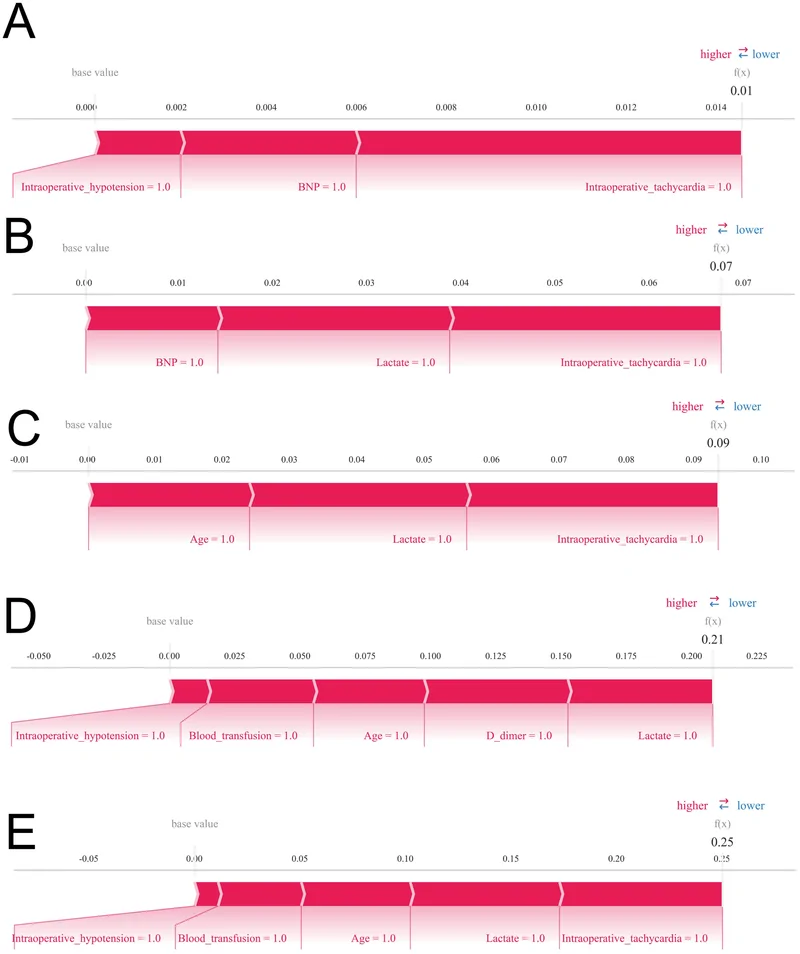
Individual-level SHAP decision analysis of representative patients. SHAP decision plots illustrate individualized risk interpretation for five representative elderly patients **(A–E)**. Each plot demonstrates the progression from baseline risk to final predicted probability.

Specifically, in patient 1 (Figure 8A), the principal contributors included intraoperative hypotension, elevated BNP, and intraoperative tachycardia, ultimately increasing the predicted risk to 0.01. In patient 2 (Figure 8B), BNP, lactate, and tachycardia jointly drove risk escalation to 0.07. In patient 3 (Figure 8C), the risk accumulation trajectory was initiated by advanced age, followed by elevated lactate and tachycardia, resulting in a predicted probability of 0.09. In contrast, patients 4 (Figure 8D) and 5 (Figure 8E) exhibited markedly higher risk scores of 0.21 and 0.25, respectively. In both cases, the risk trajectory was initially driven by intraoperative hypotension and blood transfusion, followed by the sequential accumulation of additional high-risk features, including advanced age, elevated D-dimer, increased lactate, and intraoperative tachycardia, collectively forming a synergistic risk amplification cascade.

Collectively, these SHAP-based individualized analyses reveal that intraoperative tachycardia and elevated lactate represent consistent core drivers across multiple patients, whereas intraoperative hypotension and blood transfusion exert a decisive amplifying effect in the later stages of risk accumulation.

## Discussion

This study developed and compared five machine learning models—XGBoost, RF, SVM, MLP, and KNN—to identify the most suitable algorithm for predicting postoperative cardiac arrest in elderly patients. Each model has distinct advantages: RF and XGBoost are capable of capturing complex nonlinear relationships and variable interactions; SVM performs robustly in high-dimensional classification tasks; MLP offers strong feature-learning capacity; and KNN requires no assumptions regarding data distribution (31–33). However, perioperative clinical datasets are typically characterised by high dimensionality, nonlinear structure, and heterogeneity, posing substantial challenges to model generalisability and robustness.

In the comparative performance analysis, XGBoost demonstrated superior predictive ability. ROC curve analysis showed that XGBoost achieved AUCs of 0.967 and 0.960 in the training and validation cohorts, respectively, substantially outperforming RF, SVM, MLP, and KNN, which yielded validation AUCs ranging from 0.585 to 0.759, indicating markedly stronger discriminative performance. In comparison, although RF also relies on tree-based learning and can capture nonlinear relationships, its bagging strategy primarily reduces variance and lacks the iterative error-correcting optimisation characteristic of boosting methods such as XGBoost. SVM, despite achieving a relatively high mean cross-validated AUC (0.8871 ± 0.0110), still underperformed compared with XGBoost, suggesting limitations in capturing complex feature interactions in heterogeneous clinical data. Although MLP is theoretically capable of modelling complex nonlinear patterns, it exhibited poor calibration in this study, with a Brier score as high as 0.066 vs. 0.007 for XGBoost, indicating substantial deviation between predicted probabilities and observed outcomes. KNN performed the worst overall, likely due to its reliance on distance metrics and sensitivity to the curse of dimensionality, achieving a mean validation AUC of only 0.8220 ± 0.0240.

Beyond discrimination, model stability and clinical utility are also critical evaluation metrics. Ten-fold cross-validation demonstrated that XGBoost achieved the highest mean AUC (0.8905 ± 0.0693), with a final internal dataset AUC of 0.9387, indicating strong robustness and generalisability. Learning curve analysis further confirmed that training and validation performance gradually converged with increasing sample size, with no evidence of overfitting. Moreover, XGBoost achieved the lowest Brier score and consistently yielded the highest net benefit across most threshold probabilities in decision curve analysis, whereas the MLP model even fell below the “treat-all' or “treat-none' strategies in certain ranges, indicating limited clinical applicability. Although RF, SVM, and KNN demonstrated some degree of clinical benefit, their overall performance remained inferior to XGBoost.

Importantly, the superiority of XGBoost was further validated in an independent external cohort, where it achieved an AUC of 0.971. The calibration curve closely aligned with the ideal reference line, and the bootstrap-corrected mean absolute error was only 0.002, indicating excellent external generalisability. The superior performance of XGBoost may be attributed to its gradient boosting framework, which iteratively corrects residual errors from previous models, while regularisation, tree pruning, and feature subsampling collectively reduce overfitting while capturing complex nonlinear interactions among variables (34–36). Therefore, compared with RF, SVM, MLP, and KNN, XGBoost is better suited for modelling high-dimensional heterogeneous perioperative data in elderly patients and enables more accurate risk prediction. Taken together, across discrimination, calibration, stability, and clinical net benefit, XGBoost consistently demonstrated optimal performance, further supporting its utility in risk stratification for postoperative cardiac arrest.

Although XGBoost exhibited excellent predictive performance, high accuracy alone is insufficient for guiding clinical decision-making. In recent years, with the widespread application of machine learning in medicine, model interpretability has become a central issue for clinical translation (37–39). Unlike traditional statistical models, machine learning algorithms are often regarded as “black boxes,” with limited transparency in their decision processes. Therefore, after identifying XGBoost as the optimal model, we further employed SHAP (Shapley Additive Explanations) to interpret model outputs and identify key determinants of postoperative cardiac arrest and their relative contributions (36). The results indicated that advanced age, intraoperative hypotension, intraoperative tachycardia, intraoperative blood transfusion, intraoperative hypoxaemia, and postoperative elevations in NLR, BNP, lactate, and D-dimer were the most influential predictors. These factors span the entire perioperative continuum, suggesting that postoperative cardiac arrest in elderly patients is not driven by a single determinant but rather results from the combined effects of baseline physiological vulnerability, intraoperative haemodynamic and oxygen metabolic disturbance, and postoperative physiological decompensation. Accordingly, the underlying mechanisms were further explored from these three domains to provide a theoretical basis for early identification and precise intervention.

In this study, advanced age emerged as a key risk factor identified by SHAP analysis, highlighting its central role in postoperative cardiac arrest. Ageing is associated with structural and functional cardiovascular alterations, including reduced myocardial compliance, impaired diastolic function, diminished coronary reserve, and degeneration of the cardiac conduction system. These changes substantially reduce the ability to compensate for perioperative haemodynamic fluctuations, such that even transient hypotension, blood loss, or hypoxaemia may precipitate myocardial ischaemia, arrhythmias, or circulatory collapse (1, 40–42). In addition, age-related autonomic dysfunction further impairs sympathetic–parasympathetic balance, increasing susceptibility to malignant arrhythmias and cardiac arrest.

Beyond this baseline vulnerability, intraoperative hypotension, tachycardia, blood transfusion, and hypoxaemia were also strongly associated with postoperative cardiac arrest. Although these factors differ in clinical manifestation, they collectively reflect perioperative haemodynamic instability and oxygen supply–demand imbalance, suggesting that intraoperative circulatory disturbance represents a key pathophysiological link between baseline fragility and postoperative cardiac arrest.

Intraoperative hypotension is a well-established risk factor for adverse cardiovascular events. Sustained or recurrent hypotension reduces coronary perfusion pressure, leading to myocardial ischaemia and end-organ hypoperfusion. In elderly patients with reduced coronary reserve and impaired compensatory capacity, even brief episodes of hypotension may trigger myocardial injury, acute cardiac dysfunction, and malignant arrhythmias (43–45). Mean arterial pressure below individual perfusion thresholds has been associated with increased risks of myocardial injury, acute kidney injury, and mortality, further supporting its critical role in adverse outcomes (26). Intraoperative tachycardia, as an indicator of circulatory stress, increases myocardial oxygen consumption while shortening diastole and reducing coronary perfusion, thereby exacerbating oxygen supply–demand imbalance (46, 47). When tachycardia coexists with hypotension, this imbalance is further amplified, markedly increasing the risk of myocardial ischaemia and arrhythmias. In elderly patients with limited cardiovascular reserve, this dual insult may precipitate circulatory decompensation and ultimately cardiac arrest.

Intraoperative hypoxaemia was also identified as a significant predictor. Reduced oxygen availability directly impairs myocardial and organ metabolism, promotes anaerobic metabolism, and disrupts cellular energy production. Beyond tissue hypoxia, hypoxaemia activates the sympathetic nervous system, increases pulmonary arterial pressure, and triggers arrhythmogenesis, thereby further aggravating cardiovascular stress (28, 48). Perioperative hypoxaemic events are closely associated with increased cardiovascular complications and in-hospital mortality, and elderly patients are particularly vulnerable due to reduced pulmonary reserve and pre-existing cardiopulmonary disease. In addition, intraoperative blood transfusion was identified as one of the most important predictors. Rather than being a direct causal factor, transfusion likely reflects severe blood loss, surgical complexity, and haemodynamic instability. Massive haemorrhage reduces oxygen delivery, while transfusion-related inflammatory responses, immunomodulation, and volume loading may further burden cardiac function. In elderly patients with limited cardiac reserve, rapid volume shifts may precipitate acute heart failure or circulatory collapse, thereby increasing the risk of postoperative cardiac arrest (49, 50).

Postoperative elevations in NLR, BNP, lactate, and D-dimer were also strongly associated with cardiac arrest. Although derived from different physiological systems, these biomarkers collectively reflect postoperative inflammatory activation, circulatory dysfunction, tissue hypoperfusion, and coagulation imbalance, suggesting that postoperative physiological decompensation represents a critical antecedent state. NLR reflects systemic inflammatory activation and immune dysregulation (51, 52), which may promote endothelial injury, microcirculatory dysfunction, and coagulation activation, thereby increasing the risk of arrhythmias and circulatory failure (53, 54). BNP elevation indicates ventricular wall stress and cardiac dysfunction, often resulting from perioperative fluid shifts, transfusion, and haemodynamic instability, particularly in elderly patients with limited cardiac reserve (55–57). Lactate is a well-established marker of tissue hypoperfusion and oxygen utilisation failure, with elevated levels indicating progression towards multi-organ dysfunction (58–60). D-dimer elevation reflects activation of coagulation and fibrinolysis systems (61), and in elderly patients with endothelial dysfunction and prothrombotic tendency, may contribute to thromboembolic events and microcirculatory impairment. Importantly, these pathways are interrelated, forming a self-amplifying cascade of inflammation, coagulation activation, hypoperfusion, and cardiac dysfunction that ultimately drives progression towards circulatory collapse and cardiac arrest.

An important consideration is the definition of the target population. Cardiac and major vascular surgeries were excluded to establish a relatively homogeneous cohort, as these procedures involve highly specific surgical and hemodynamic conditions, such as cardiopulmonary bypass, vascular clamping, substantial blood loss, and marked hemodynamic fluctuations, which may lead to distinct mechanisms of IHCA and perioperative management pathways. Similarly, patients with major pre-existing cardiovascular abnormalities, markedly abnormal preoperative cardiac biomarkers, or severe hepatic or renal dysfunction were excluded because these conditions often require substantially different preoperative assessment, anesthetic planning, intraoperative monitoring, and postoperative care. Therefore, their clinical management and outcomes may be strongly influenced by disease-specific pathways rather than the general perioperative physiological disturbances targeted by our model. Our model was instead designed to identify elderly non-cardiac surgical patients without major baseline organ dysfunction whose IHCA risk increases substantially following perioperative physiological stress and early postoperative deterioration. We acknowledge that these criteria limit the generalizability of the model to patients undergoing cardiac or major vascular surgery and those with severe baseline cardiovascular or organ dysfunction. Future studies should develop and validate dedicated models for these high-risk populations.

Despite integrating comprehensive perioperative variables and performing independent external validation, several limitations should be acknowledged. Because the model incorporated early postoperative laboratory measurements, risk prediction was performed after surgery, when the first laboratory assessment within 24 h became available, and therefore the model was not intended to guide preoperative optimization or immediate postoperative management. In addition, the predefined age threshold may not fully reflect biological age, frailty, or physiological reserve, and the applicability of the model to younger but clinically vulnerable patients remains uncertain. The model also did not incorporate continuous intraoperative physiological trajectories or postoperative vital signs, which may have limited its ability to capture the magnitude, variability, cumulative burden, and temporal patterns of physiological deterioration. Furthermore, our analysis focused on the index surgery and did not account for recent surgery or complex prior hospitalization, which may provide additional prognostic information. The availability and routine clinical use of certain postoperative biomarkers, such as BNP and D-dimer, also vary across healthcare systems, potentially limiting the broader applicability of the model. Although stratified sampling, 10-fold cross-validation, and independent external validation were used, the relatively small number of IHCA events may still have affected model stability, and repeated stratified cross-validation or bootstrap validation was not performed. In addition, PR-AUC, PPV, NPV, calibration intercept and slope, and threshold-specific sensitivity were not included in the original evaluation and should be assessed in future validation studies, particularly given the low incidence of IHCA. Finally, the retrospective design, potential selection bias related to missing data and exclusion of patients who developed IHCA before postoperative laboratory assessment, and the possibility of residual instability during feature selection should be considered. Future prospective multicentre studies with larger and more diverse populations are warranted to further evaluate the robustness, transportability, and clinical utility of the model.

## Conclusion

In conclusion, using multicentre clinical data, this study developed and validated a machine learning–based predictive model for postoperative cardiac arrest in elderly patients. Compared with RF, SVM, MLP, and KNN, XGBoost demonstrated superior discrimination, calibration, stability, and clinical net benefit, with excellent performance in external validation. SHAP analysis identified advanced age, intraoperative hypotension, intraoperative tachycardia, intraoperative blood transfusion, intraoperative hypoxaemia, and postoperative elevations in NLR, BNP, lactate, and D-dimer as key determinants of postoperative cardiac arrest. These predictors span preoperative vulnerability, intraoperative physiological disturbance, and postoperative decompensation, underscoring that postoperative cardiac arrest is a multifactorial, multistage pathophysiological process.

## Data Availability

The original contributions presented in the study are included in the article/Supplementary Material, further inquiries can be directed to the corresponding authors.

## Supplementary material

Supplementary Figure S1 Comparison of the Discriminatory Performance of Conventional Risk Stratification Tools. (A) ROC curve for ASA. (B) ROC curve for RCRI.
